# Father-Child Conflict and Chinese Adolescent Depression: A Moderated Mediation Model

**DOI:** 10.3389/fpsyg.2021.723250

**Published:** 2021-10-07

**Authors:** Cong Peng, Jianwen Chen, Huifen Wu, Yan Liu, Youguo Liao, Yuqin Wu, Xintong Zheng

**Affiliations:** ^1^School of Education, Huazhong University of Science and Technology, Wuhan, China; ^2^Hunan Railway Professional Technology College, Zhuzhou, China; ^3^Mental Health Education Center, Minjiang University, Fuzhou, China; ^4^School of Foreign Languages, Zhongnan University of Economics and Law, Wuhan, China

**Keywords:** father-child conflict, regulatory emotional self-efficacy, depression, adolescent, gender

## Abstract

To investigate the effects of father-child conflict and regulatory emotional self-efficacy (RESE) on Chinese adolescent depression, 654 middle-school students were measured. The results showed that: (1) Father-son conflict was significantly lower than father-daughter conflict, girls’ depression was significantly higher than that of boys, and boys’ RESE and self-efficacy in regulating negative emotions (NEG) were significantly higher than that for girls, but there was no significant difference between boys and girls in self-efficacy in expressing positive emotions (POS). (2) Father-child conflict was significantly positively associated with Chinese adolescent depression. Father-child conflict was negatively correlated with RESE, and its two dimensions. Both POS and NEG played a partial mediating role in the relationship between father-child conflict and adolescent depression. (3) Gender only regulated the relationship between NEG and adolescent depression. Compared to boys, girls are more affected by depression at the low level of NEG.

## Introduction

Depression is one of the most common emotional disorders in adolescence, and its symptoms are the precursor of psychological diseases, which usually begin in adolescence and continue to adulthood (Andersen and Teicher, 2008; Auerbach et al., 2014; McLeod et al., 2016). A recent study found that the detection rate of adolescent depression in the United States is 12.8–18.1% (Lu, 2019), while a national mental health survey in China showed that the detection rate of adolescent depression is around 37% (Chen et al., 2012). Depression can lead to a series of psychological and social adaptation problems, such as academic performance decline (Liu et al., 2017), social withdrawal (Hamasaki et al., 2020), dangerous sexual behavior (Foley et al., 2019), substance abuse (Bentley et al., 2021), internet addiction (Chi et al., 2019), or even suicide (Parker and Ricciardi, 2019). Therefore, it is important to investigate the influencing factors of adolescent depression.

Good interpersonal relationships help alleviate and suppress depression, while bad relationships can cause and maintain depression (Rudolph et al., 2008). The parent-child relationship is the most important interpersonal relationship among adolescents and the core family environment factor, that affects the growth of adolescents (Branje, 2018). There are two dimensions of positive and negative interaction in parent-child relationships: support and conflict (Laursen and Collins, 2009). From pre-puberty to early and middle adolescence, the perceived support of parents gradually decreases and the conflict between parents and adolescents increases (Koepke and Denissen, 2012; Hazel et al., 2014). Many studies have shown that parent-child conflict and conflict in a family environment are important risk factors for depression among adolescents (Branje et al., 2010; Liu et al., 2011; Smokowski et al., 2014; Withers et al., 2016). However, to date, most studies on parent-child relationships have primarily examined the impact of mothers on adolescents (Allen et al., 2003; Schudlich et al., 2004; Branje, 2018), or combined the effects of father and mother to investigate the effects of parent-child relationship on adolescent adaptation (Helsen et al., 2000; Wu and Zhang, 2004). The same applies to the study of parent-child conflict. Many studies do not distinguish father-child conflict from mother-child conflict, but generally name it as parent-child conflict. In fact, fathers are known as “forgotten contributors to the development of children” (Lamb, 1975). Research shows that fathers play an irreplaceable role in the growth of children, and the father-child relationship is closely related to their mental health of children (Brotherson et al., 2003; Bornovalova et al., 2014; Rodríguez et al., 2019), as well as their emotional health of children (Mallers et al., 2010; Teel et al., 2016). A meta-analysis of 34 studies found that fathers play a unique role in parenting that differs from that of mothers, and there is an association between fathering and the outcome variables held across social measurements, psychological indicators, and academic achievement (Jeynes, 2016).

Fathers’ roles might actually grow in importance during adolescence as physical care-taking declines in prominence (Phares and Compas, 1992). The mother’s “inputs” are not consistently associated with their children’s development indices after entering secondary school, whereas father’s inputs are so correlated (Lamb and Lewis, 2010). It is, therefore, necessary to investigate the influence of paternity on adolescent depression. Teenagers are in a period of growth rebellion, and the conflicts between parents and children increases (Hazel et al., 2014; Yan et al., 2019). At the same time, the nature of parent-child conflict is also manifested in the transition from poor parent-child attachment in childhood to parent-child conflict in the process of separation-individualization in adolescence. At this stage, the participation of fathers increases, and the conflict between father and child becomes increasingly prominent. Not surprising, father-child conflict has been found to be positively associated with adolescent depression (Sallinen et al., 2007; Delgado et al., 2013; Brouillard et al., 2018). Even if the mother-child conflict and parental conflict are controlled, the relationship between father-child conflict and adolescent depression is still significant (Cole and McPherson, 1993). Although there are some evidences regarding the association between father-child conflict and adolescent depression, it is not known yet through which pathway father-child conflict influence adolescent depression. Thus, the present study examined one potentially important mediating factor between them - Regulatory emotional self-efficacy.

Regulatory emotional self-efficacy (RESE) is the capability of individuals to effectively adjust their emotional state (Bandura et al., 2003; Caprara et al., 2008). Previous studies have demonstrated that RESE is an important determinant of adolescents’ emotional health (Caprara et al., 2008, 2010; Compas et al., 2017; De Castella et al., 2018; Di Giunta et al., 2018). In addition, adolescent’s RESE was also influenced by parent-child relationship. According to the family model of emotion regulation, adolescents learn emotion regulation by observing parents’ emotional performance and by the parent-child interaction (Morris et al., 2007; Gresham and Gullone, 2012). The increase of frequency or intensity of parent-child conflicts can reduce emotional regulation self-efficacy (Huang et al., 2015). Hence, RESE could be an important path for parent-child conflict, which affects adolescent depression.

Furthermore, the influence of parent-child conflict and RESE on adolescent depression may be regulated by the gender. First, a longitudinal study examined the trajectories of parent-child relationship from Grades 1 to 6 and their associations with child depressive symptoms. The results indicated that girls who experienced a slower increase in father-child conflict, and a slower decrease in father-child closeness, were at lower risk for depressive symptoms. Boys who experienced a slower increase in mother-child conflict were at lower risk for depressive symptoms (Yan et al., 2019). The predictive link between parent-child relationship quality and depressive symptoms may vary by the gender of the adolescent (Brouillard et al., 2018). Conflict with parents was a stronger predictor of depressive symptoms for early adolescent girls than for boys, explaining 19 and 6% of the variance, respectively (Jenkins et al., 2002). These findings indicate that understanding parent-child relationship impact on adolescent mental health requires distinctions according to both the children and parents’ gender (Smith et al., 2019; Ma et al., 2020). Second, gender may also moderate RESE and adolescent depression. Research has found that females had greater general difficulties with emotion regulation than males (Malesza, 2019). Compared to males, females have stronger negative emotional susceptibility (Mak et al., 2009). Depression is often related to the individual’s cognition and ability to regulate their negative emotions (Caprara et al., 2010). Therefore, how and to what extent does the father-child conflict affect adolescent depression? Is there a gender difference? This is undoubtedly worthy of further study.

In addition, fathering and father-child relationship were profoundly influenced by the cultures to which they belong (Seward and Stanley-Stevens, 2014). Chinese families and fathers differ to some degree from European and American families in terms of parenting traditions, family structure, and gender roles (Li and Lamb, 2013). Nevertheless, although previous studies have focused on the relationship between father-child conflict and adolescent depression in western cultures, little is known about how father-child relationship quality, especially conflict, is related to adolescent depression in Chinese culture. Thus, addressing the gap in the research of father-child conflict’s impact on adolescent depression, this study first aims to answer the question of how the father-child conflict is directly related to Chinese adolescent depression. Second, indirect effects of father-child conflict on adolescent depression *via* the mechanism of RESE were tested. Third, whether the direct and indirect effects are moderated by gender of adolescents was also examined.

### Father-Child Conflict and Adolescent Depression

Based on the attachment theory (Bowlby, 1973), when teenagers have a close relationship with their parents, they show a positive development trend and are less likely to be depressed. Conversely, a poor parent-child relationship makes them prone to depression as they lack emotional support from parents when they are faced with emotional distress. Feeling accepted by parents can improve teenagers’ self-efficacy and help them to avoid depression (Windle, 1992). Some cross-sectional studies have found that the relationship between father-child conflict and depression was significantly positive (Videon, 2005; Delgado et al., 2013). Even if the mother-child conflict and parental conflict are controlled, the relationship between father-child conflict and adolescent depression is still significant (Cole and McPherson, 1993). Positive father-child communication can predict depression at a lower level in adolescents (Bireda and Pillay, 2018). In addition, the results of longitudinal studies showed that the conflict between father and child can significantly predict adolescent depression (Sallinen et al., 2007; Brouillard et al., 2018). Moreover, a cross-cultural study of four countries also showed that the impact of father-child conflict on adolescent depression was consistent in cross-cultural and cross- development stages (Vazsonyi and Belliston, 2006). Based on the above findings, this study proposed that the father-child conflict is significantly related to depression in adolescents (Hypothesis 1). Although there are some evidences regarding the association between father-child conflict and adolescent depression, it is not known yet through which pathway father-child conflict affect adolescent depression. Therefore, this study aimed to answer the question of how the father-child conflict affects adolescents’ depression and “under what conditions the effect is more significant.” This will not only help us to understand the effect path of father-child conflict on adolescent depression, but will also bring enlightenment significance to prevent and reduce depression in adolescents.

### The Mediating Effect of Regulatory Emotional Self-Efficacy

Regulatory emotional self-efficacy (RESE) is the capability of individuals to effectively adjust their emotional state, including expressing positive emotional self-efficacy (POS) and managing negative emotional self-efficacy (NEG). The former refers to the ability to express positive emotions freely, while the latter refers to the ability to ameliorate negative emotional states (Bandura et al., 2003; Caprara et al., 2008; Dou et al., 2013). As an extension of the concept of self-efficacy, RESE has become a new research topic in recent years and has a close relationship with mental health (Tang et al., 2010). It provides a more specific and clear perspective for us to better understand the effect path of father-child conflict on adolescent depression. First, the level of RESE is an important factor in predicting depression (Caprara et al., 2010; De Castella et al., 2018). Some studies have shown that RESE not only affects depression in the short term but also has a long-term effect over time (Caprara et al., 2002). In addition, a longitudinal study showed that self-efficacy in regulating negative emotions was highly correlated with adolescent depression (Hai et al., 2019). Individuals with high emotional regulation efficacy have the confidence and belief in their emotional regulation ability, so they will experience more positive emotions and be less affected by negative emotions, and therefore, have lower levels of depression. Conversely, when adolescents’ RESE is at a low level, they will not be able to regulate their emotions effectively, and negative emotions are not effectively relieved which can easily induce depression. Therefore, lower levels of RESE can lead directly to depression (Bandura et al., 2003; Compas et al., 2017).

In addition, parent-child conflict is an important influencing factor of individual RESE. According to the family model of emotion regulation, parent-child interaction is an important influencing factor. Adolescents learn by observing parents’ emotional performance and by the parent-child interaction (Morris et al., 2007; Gresham and Gullone, 2012). Herd et al. (2020) found that the quality of the parent-adolescent relationship is an important factor in predicting adolescents’ emotion regulation. An empirical study by Huang et al. (2015) also found that the increase of frequency or intensity of parent-child conflicts (including father-child conflict and mother-child conflict) can reduce emotional regulation self-efficacy, while Gresham and Gullone (2012) found that parents’ emotional warmth and family intimacy can positively predict emotional regulation self-efficacy. Hence, good parent-child interaction can improve RESE, while higher parent-child conflict may reduce it, thus affecting the effect of emotion regulation, leading to adverse emotional distress. Therefore, RESE is an important path for parent-child conflict, which affects adolescent depression.

In conclusion, RESE could play a mediating role in the relationship between father-child conflict and adolescent depression (Hypothesis 2). However, most previous studies combined father-child conflict with mother-child conflict and rarely investigated the impact of father-child conflict on adolescent depression. At present, the empirical research data on this hypothesis is quite scarce, and there is no research on the mediating role of emotional regulation self-efficacy (RESE) in the relationship between father-child conflict and adolescent depression.

### The Moderating Effect of Gender

The above mediation process may also be regulated by gender. First, the relationship between father-child conflict and adolescent depression may be regulated by gender (Nolen-Hoeksema and Girgus, 1994; Branje et al., 2010). Adolescent girls have a higher emotional response to interpersonal stress than boys (Nolen-Hoeksema, 2001; Zahn-Waxler et al., 2008), and the depression of adolescent girls has been found to be significantly higher than that of boys (Angold et al., 2002; Ma et al., 2020). Research found that the effect of parent-child conflict on adolescent depression is more intense among girls (Jenkins et al., 2002). Studies of Chinese adolescent samples also showed that close proximity to fathers predicts lower depression risk in girls (Ma et al., 2020). In the early and middle adolescence, father-child conflict is only associated with depression of girls in some grades. The relationship between father-child conflict and depression is regulated by gender (Liu et al., 2011).

Second, an individual’s gender is an important variable to regulate emotional regulation self-efficacy and depression. Research has found that females had greater general difficulties with emotion regulation than males (Malesza, 2019), while the latter had a more robust sense of personal efficacy in dealing with negative affect than did females, especially at earlier ages (Caprara et al., 2003). Hence, because of the high correlation between RESE and depression, females are more likely to be affected by a decrease of RESE.

Third, fathering and the father-child relationship are profoundly influenced by culture (Seward and Stanley-Stevens, 2014). Traditional Chinese fathering is considered authoritarian, emotionally reserved and affectively distant. Children are supposed to be compliant, respectful, and filial toward their parents, especially their fathers (Li and Lamb, 2013). Under the influence of the patrilineal system and patriarchal ideology, the father-son relationship often outweighs the spousal relationship, and sons are regarded as more important than daughters (Chuang et al., 2013). Although contemporary Chinese fathers are more likely to seek increasing involvement in their children’s lives than before (Chuang et al., 2013; Li and Lamb, 2013), Confucianism and traditional Chinese culture remain influential on Chinese parenthood (Ishii-Kuntz, 2015). For example, parents have different expectations for the role of male and female children. According to Chinese traditional cultures, they encourage their daughters to be dependent and obedient, and their sons to be autonomous and competitive. Many fathers do not treat their daughters as independent individuals and prefer their daughters to be more obedient than they actually are (Xu, 2016). Based on this, we infer that when Chinese adolescent girls have conflicts with their father, they are more likely to make compromises and suppress negative emotions toward their father, which will affect their RESE.

Therefore, the study hypothesizes that gender may play a moderating role in the direct effect of father-child conflict on adolescent depression, and the second half path of the mediating effect may be moderated by gender (Hypothesis 3).

### Current Research

**FIGURE 1:**
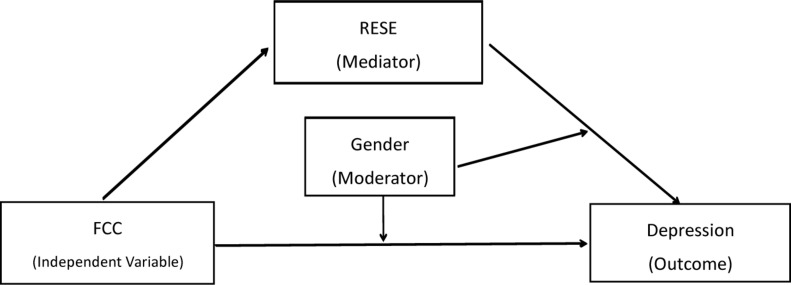
The proposed moderated mediation model. RESE: regulatory emotional self-efficacy; FCC: father-child conflict.

On the basis of attachment theory and family model of emotion regulation, this study constructed a mediating model with regulation (see Figure 1), and examined the relationship between father-child conflict, RESE, gender, and adolescent depression. Specifically, this study aimed at investigating the mediating (RESE) and moderating (gender) effects of father-child conflict on adolescent depression, so as to clarify the possible path of father-child conflict affecting adolescent depression and its group differences, and provide empirical support and theoretical guidance for the prevention and reduction of adolescent depression in China.

## Materials and Methods

### Participants

The participants were 677 adolescents recruited from three middle schools of Hubei Province, China. A group test was carried out. A total of 677 paper questionnaires were distributed and 654 valid questionnaires were collected, with an effective rate of 96.6%. The age of the subjects ranged from 11 to 16 years old, with an average age of 12.94 ± 1.11. There were 364 male students (55.7%) and 290 female students (44.3%). The participants consisted of 371 (56.7%) in Grade One, 187 (28.6%) in Grade Two and 96 (14.7%) in Grade Three.

### Measures

#### Father-Child Conflict Scale

The conflict behavior scale (Prinz et al., 1979; Robin and Foster, 1989) was used to measure the cognition of children and adolescents on the degree of conflict and negative communication between them and their parents, as reported by the children and adolescents themselves. In this study, the Chinese version of father-child conflict behavior scale (Yin et al., 2013) including 20 items was used (i.e., “My dad always seems to be complaining about me.” and “At least three times a week, we get angry at each other.”). There were only two answers for each item: “yes” or “no.” A high score means high father-child conflict. The internal consistency coefficient was 0.890 and the test-retest reliability was 0.830 after an interval of 4 weeks. In this study, confirmatory factor analysis (CFA) indicated that RESE has accepted constructive validity (χ^2^/*df* = 4.003, CFI = 0.863, TLI = 0.846, SRMR = 0.050, and RMSEA = 0.068). The Cronbach’α of the scale is 0.875. This indicates that the reliability and validity of the scale are good.

#### Regulatory Emotional Self-Efficacy Scale

The revised Chinese version (Dou et al., 2013; Wang et al., 2013) of the RESE Scale (Caprara et al., 2008) was used in this study. The scale consists of 17 items, which were divided into two dimensions: perceived self-efficacy in expressing positive affect (POS) (i.e., “I can express enjoyment freely at parties.”) and perceived self-efficacy in managing negative affect (NEG) (i.e., “I can keep from getting dejected when I am lonely.”). In this study, participants rated each item on a 5-point scale ranging from 1 = very inconsistent to 5 = very consistent, with higher scores indicating a higher level of RESE. Confirmatory factor analysis (CFA) indicated that RESE has accepted constructive validity (χ^2^/*df* = 3.254, CFI = 0.918, TLI = 0.902, SRMR = 0.071, and RMSEA = 0.059). The Cronbach’α of the scale was 0.897, and 0.856, and 0.877 for each dimension. This indicates that the scale has good reliability and validity.

#### Center for Epidemiological Studies on Depression Scale

The center for epidemiological studies on depression (CES-D) scale (Radloff, 1977) was used to measure depressive emotion or mood. It is widely used in the screening of depressive symptoms in the general population and has good applicability in Chinese adolescents. The Cronbach’s α of the scale was 0.880 (Chen et al., 2009). There are 20 items in the scale (i.e., “I was upset by things that don’t normally upset me.”). The participants rated each item on a 4-point scale ranging from 0 = rarely or none of the time (less than 1 day) to 3 = most or all of the time (5–7 days), with higher scores indicating a higher level of depression. In this study, confirmatory factor analysis (CFA) indicated that RESE has accepted constructive validity (χ2/*df* = 3.661, CFI = 0.910, TLI = 0.896, SRMR = 0.045, and RMSEA = 0.064). The Cronbach’α of the scale is 0.890. This indicates that the reliability and validity of the scale are good.

### Control Variables

In addition to the main variables of interest, we used other child and family characteristics to control for possible biasing factors. Age and grade may be correlated with the trajectories of parent-child conflict among adolescents (Smetana et al., 2004; Mastrotheodoros et al., 2020). The associations among adolescent emotional and behavioral outcomes and father-child relationship qualities vary with symptom type and family structures (Little et al., 2019). The change of family structure could profoundly affect the nature of the father-child relationship (Hornstra et al., 2020). Therefore, we controlled age, grade, and family structure as covariates in the models. In this study, family structure was coded as a category variable (1 = intact family; 2 = single-parent family; 3 = reconstituted family; 4 = other types).

### Procedure

The data were collected in middle-school classrooms without the presence of teachers between October and November in 2020. Written informed consent to participate in this study was provided by the participants’ legal guardian/next of kin. The research protocol was approved by the Ethical Committee of the School of Education of Huazhong University of Science and Technology. Prior to the survey, the students were informed that their participation was voluntary and they could withdraw from the study at any time. Trained post-graduate students completed the distribution and recycling of questionnaires in accordance with the standard procedures and addressed the students’ questions immediately.

### Data Analysis

SPSS version 23.0 was used for descriptive statistics, an independent sample *t*-test and correlation analysis. The independent sample *t*-test was used to analyze the score differences of adolescents of different genders in each research variable, and the Pearson correlation was used to analyze the correlation coefficient of each variable. Then the PROCESS macro program (Hayes, 2012) was conducted to test the mediating and moderating effects, controlling for the variables of age, grade, and family structure. First, model 4 (it is a simple mediating model) in PROCESS macro program for SPSS was used to test the mediating effect of RESE, between father-child conflict and adolescent depression. The bias adjusted percentile bootstrap method was used to test the mediating effect. Five thousand samples were selected to estimate the 95% confidence interval of the mediating effect. If the bootstrap 95% confidence interval did not contain zero, we could be reasonably certain that the mediating effect could be found. Then model 15 (it is assumed that the direct path and the second half path of the mediating model are moderated, consistent with the theoretical model of this study) in the PROCESS macro program for SPSS would be used to test the moderated mediation model of father-child conflict on adolescent depression.

## Results

As all the data in this study were derived from adolescents’ self-reports, it is possible that the common-method bias might occur. To negate this possibility, Harman’s single factor Test was used to test the common-method bias. The results of non-rotating principal component factor analysis showed that there were 11 factors with characteristics greater than 1, and the variation explained by the first factor was 11.75% (less than 40% of the critical value), indicating that there was no serious common-method bias in this study.

### Preliminary Analysis

Taking gender as the independent variable (0 = male; 1 = female), father-child conflict, total score of RESE and each dimension score, and depression as the dependent variable, an independent sample *t*-test was conducted. The results showed that there were significant differences in father-child conflict, RESE and perceived self-efficacy in managing negative affect (NEG) among adolescents of different genders. The father-child conflict of boys was significantly lower than that of girls (*t* = −2.45, *P* < 0.05), boys’ RESE and NEG were significantly higher than girls’ (*t* = 2.37,*P* < 0.05;*t* = 3.32,*P* < 0.001). The depression score of female students was significantly higher than that of male students (*t* = −2.85, *P* < 0.01), but there was no significant difference in self-efficacy in expressing positive emotions (POS) between male and female students (see Table 1).

**TABLE 1:** *T*-testing of scores between males and females on the main study variables (*N* = 654).

	**FCC**	**RESE**	**POS**	**NEG**	**Depression**
Male	0.34 ± 0.25	3.62 ± 0.76	3.81 ± 0.91	3.44 ± 0.83	0.94 ± 0.55
Female	0.39 ± 0.27	3.48 ± 0.76	3.74 ± 0.93	3.22 ± 0.82	1.07 ± 0.62
*t*	−2.45*	2.37*	0.95	3.32***	−2.85**

*P < 0.05, **P < 0.01, ***P < 0.001; FCC: father-child conflict; RESE: regulatory emotional self-efficacy; POS: self-efficacy in expressing positive affect; NEG: self-efficacy in managing negative affect.

The mean, standard deviation, and correlation coefficient of each variable are listed in Table 2. The results showed that father-child conflict was positively correlated with depression (*Ps* < 0.01), and negatively correlated with RESE, POS, and NEG (*Ps* < 0.01); depression was negatively correlated with RESE, POS, and NEG (*Ps* < 0.01); gender was significantly correlated with father-child conflict (*Ps* < 0.05), RESE (*Ps* < 0.01), POS (*Ps* < 0.05), and depression (*Ps* < 0.01).

**TABLE 2:** The correlation of the main study variables (*N* = 654).

**Variables**	***M* ± *SD***	**1**	**2**	**3**	**4**	**5**	**6**
1 FCC	0.36 ± 0.26	1					
2 POS	3.78 ± 0.92	−0.33**	1				
3 NEG	3.34 ± 0.83	−0.32**	0.52**	1			
4 RESE	3.56 ± 0.76	−0.37**	0.89**	0.86**	1		
5 Depression	1.00 ± 0.59	0.47**	−0.40**	−0.40**	−0.46**	1	
6 Gender	0.44 ± 0.50	−0.10*	0.04	0.13**	0.09*	−0.11**	1

*P < 0.05, **P < 0.01; Gender was a binary variable (0 = male; 1 = female); FCC: father-child conflict; RESE: regulatory emotional self-efficacy; POS: self-efficacy in expressing positive affect; NEG: self-efficacy in managing negative affect.

### Testing for Mediation Effect

Table 2 shows that father-child conflict and depression, father-child conflict and RESE (POS and NEG), and RESE (POS and NEG) and depression are significantly correlated. Independent variables are significantly associated with mediating variables and meet the conditions of a mediating effect test. In this study, the bias adjusted percentile bootstrap method was used to test the mediating effect. Five thousand samples were selected to estimate the 95% confidence interval of mediating effect, and model 4 (a simple mediating model) in SPSS macro compiled by Hayes (2012) was used to test it.

First, under the condition of controlling age, grade, and family structure, taking POS as a mediating variable, father-child conflict as a predictive variable and depression as a dependent variable, the regression analysis on the mediating effect of POS (see Table 3) indicated that father-child conflict had significant positive prediction effect on depression (*B* = 0.47, *t* = 12.53, *P* < 0.001). When the mediating variable was added, the direct prediction effect of father-child conflict on depression was still significant (*B* = 0.38, *t* = 10.01, *P* < 0.001). Father-child conflict had a significant negative prediction effect on POS (*B* = −0.33 *t* = −7.90, *P* < 0.001), and POS had a significant negative prediction effect on depression (*B* = −0.27, *t* = −6.62, *P* < 0.001). In addition, Table 4 shows the mediating effect of father-child conflict on depression. The bootstrap 95% confidence interval of the mediating effect of POS does not contain 0 value. The direct effect (0.38) and mediating effect (0.09) account for 80.85 and 19.15% of the total effect (0.47), respectively. This indicates that POS has a partial mediating effect between father-child conflict and depression (see Table 4).

**TABLE 3:** Results for the mediation effect of POS.

**Regression equation (*N* = 654)**		**Fitting indicators**			**Coefficient significance**	
**Outcomes**	**Predictors**	***R***	***R* ^2^**	***F***	**β**	***t***
Depression		0.48	0.23	40.96***		
	Age				0.06	1.29
	Grade				0.05	0.82
	Family structure				–0.07	–0.87
	FCC				0.47	12.53***
POS		0.34	0.12	16.33***		
	Age				–0.01	–0.09
	Grade				–0.05	–0.74
	Family structure				0.10	0.93
	FCC				–0.33	−7.90***
Depression		0.54	0.29	41.59***		
	Age				0.06	1.38
	Grade				0.04	0.65
	Family structure				–0.09	–1.23
	POS				–0.27	−6.62***
	FCC				0.38	10.01***

***P < 0.001; FCC: father-child conflict; POS: self-efficacy in expressing positive affect; Family structure was a category variable (1 = intact family; 2 = single-parent family; 3 = reconstituted family; 4 = other types).

**TABLE 4:** The Estimates of total, direct and indirect effects of the model (POS as mediating variable).

	**Effect**	**Boot SE**	**Boot LLCI**	**Boot ULCI**	**Relative effect value**
Total effect	0.47	0.04	0.39	0.54	
Direct effect	0.38	0.04	0.30	0.45	80.85%
Indirect effect of POS	0.09	0.02	0.06	0.12	19.15%

Then, under the condition of controlling age, grade, and family structure, taking NEG as a mediating variable, father-child conflict as a predictive variable and depression as a dependent variable, the regression analysis on the mediating effect of NEG (see Table 5) indicated that father-child conflict has a significant positive prediction effect on depression (*B* = 0.47, *t* = 12.53, *P* < 0.001), When the mediating variable was added, the direct prediction effect of father-child conflict on depression was still significant (*B* = 0.38, *t* = 10.25, *P* < 0.001). Father-child conflict had a significant negative predictive effect on NEG (*B* = −0.32, *t* = −8.08, *P* < 0.001), and NEG had a significant negative prediction effect on depression (*B* = −0.27, *t* = −6.95, *P* < 0.001). In addition, Table 6 shows the mediating effect of father-child conflict on depression. The bootstrap 95% confidence interval of the mediating effect of NEG did not contain 0 value. The direct effect (0.38) and mediating effect (0.09) account for 80.85 and 19.15% of the total effect (0.47), respectively. This indicates that NEG also had a partial mediating effect between father-child conflict and depression.

**TABLE 5:** Results for the mediation effect of NEG.

**Regression equation (*N* = 654)**		**Fitting indicators**			**Coefficient significance**	
**Outcomes**	**Predictors**	***R***	***R* ^2^**	***F***	**β**	***t***
Depression		0.48	0.23	40.96***		
	Age				0.06	1.29
	Grade				0.05	0.82
	Family structure				–0.07	–0.87
	FCC				0.47	12.53***
NEG		0.35	0.12	21.43***		
	Age				–0.07	–1.81
	Grade				–0.11	–1.73
	Family structure				0.13	1.54
	FCC				–0.32	−8.08***
Depression		0.54	0.29	42.67***		
	Age				0.04	0.99
	Grade				0.02	0.40
	Family structure				–0.03	–0.38
	NEG				–0.27	−6.95***
	FCC				0.38	10.25***

***P < 0.001; FCC: father-child conflict; NEG: self-efficacy in managing negative affect; Family structure was a category variable (1 = intact family; 2 = single-parent family; 3 = reconstituted family; 4 = other types).

**TABLE 6:** The Estimates of total, direct and indirect effects of the model (NEG as mediating variable).

	**Effect**	**Boot SE**	**Boot LLCI**	**Boot ULCI**	**Relative effect value**
Total effect	0.47	0.04	0.39	0.54	
Direct effect	0.38	0.04	0.31	0.45	80.85%
Indirect effect of NEG	0.09	0.02	0.06	0.12	19.15%

### Testing for Moderated Mediation

Both POS and NEG had a partial mediating effect between father-child conflict and depression. Then, the model 15 (it is assumed that the direct path and the second half path of the mediating model are moderated, consistent with the theoretical model of this study) in SPSS macro compiled by Hayes (2012) was used to test the moderated mediation model of father-child conflict affecting adolescent depression.

**FIGURE 2:**
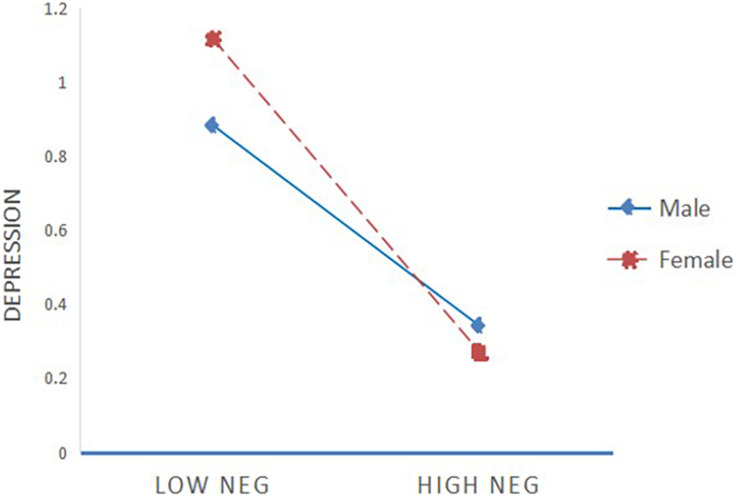
Moderating effect of gender between NEG and depression.

First, the moderated mediation model (POS as a mediating variable) of father-child conflict affecting adolescent depression was tested. The results showed that gender had no moderating effect on the direct prediction of depression caused by the father-child conflict (*B* = 0.08, *t* = 1.06, *P* > 0.05), and gender had no moderating effect on the direct prediction of POS on depression (*B* = −0.15, *t* = −1.84, *P* > 0.05). Second, the moderated mediation model (NEG as a mediating variable) of father-child conflict affecting adolescents’ depression was tested. The results (see Table 7) showed that gender had no moderating effect on the direct prediction of depression by the father-child conflict (*B* = 0.12, *t* = 1.70, *P* > 0.05), but gender can moderate the prediction effect of NEG on depression (*B* = −0.15, *t* = −1.97, *P* < 0.05). A further simple slope analysis demonstrated that the predictive effect of NEG on depression of girls (*simple slope* = −0.47, *t* = −8.27, *P* < 0.001) was higher than that of boys (*simple slope* = −0.33, *t* = −7.14, *P* < 0.001). This shows that, compared to boys, girls are more affected by depression at the low level of NEG (see Figure 2).

**TABLE 7:** Testing the moderated mediation effect of father-child conflict on adolescent depression.

**Regression equation (*N* = 654)**		**Fitting indicators**			**Coefficient significance**	
**Outcomes**	**Predictors**	***R***	***R* ^2^**	***F***	**β**	***t***
NEG		0.35	0.12	21.43***		
	Age				–0.07	–1.81
	Grade				–0.11	1.73
	Family structure				0.13	1.54
	FCC				–0.32	−8.08***
Depression		0.55	0.31	31.53***		
	Age				0.05	1.07
	Grade				0.02	0.33
	Family structure				–0.04	–0.46
	NEG				–0.27	−6.88***
	FCC				0.38	10.45***
	NEG × Gender				–0.15	−1.97*
	FCC × Gender				0.12	1.70

*P < 0.05, ***P < 0.001; FCC: father-child conflict; NEG: self-efficacy in managing negative affect; Family structure was a category variable (1 = intact family; 2 = single-parent family; 3 = reconstituted family; 4 = other types).

## Discussion

### The Mediating Role of Regulatory Emotional Self-Efficacy

The results show that RESE (POS and NEG) has a partial mediating effect in the relationship between father-child conflict and adolescent depression. Father-child conflict can not only directly affect the depression of adolescents but also indirectly through RESE. This is consistent with both Hypothesis 1 and 2. First, the relationship between father-child conflict and adolescent depression was significantly positive, which was consistent with previous studies (Sheeber et al., 1997; Liu et al., 2011; Yan et al., 2019). This indicated that father-child conflict is a direct factor affecting adolescent depression. The father plays an important role in teenagers’ emotional health. Secondly, there is a significant negative correlation between RESE and adolescent depression, that is, the higher the level of self-efficacy of emotional regulation, the less likely that depression will occur. This could be because that teenagers with better levels of self-efficacy of emotional regulation can change their negative emotional state in the face of bad emotions so as to reduce the risk of depression caused by the long-term failure to release bad emotions (Wu et al., 2015), which further illustrates the important role of adolescents’ self-efficacy of emotional regulation.

When there is conflict between adolescents and fathers, their self-efficacy of emotional regulation will be negatively affected, which increases the possibility of depression. Based on the father vulnerability hypothesis (Cummings et al., 2010), the father-child relationship (compared with the mother-child relationship) is more likely to be affected by other factors such as spouse, marital relationship, and social culture (Doherty et al., 1998; Jessee and Adamsons, 2018; Mastrotheodoros et al., 2020). Adolescents experience more volatile relations with fathers than with mothers (Videon, 2005). When the instability of father-child relationship is relatively high, the conflict will be more intense in the case of father-child conflict (Wang and Zhang, 2007). Therefore, conflict between adolescents and fathers may have serious consequences. The possibility of conflict with fathers in an attempt to change their mind or opinion is less than that of mothers (Fang and Dong, 1998), which makes the relationship between fathers and children more difficult to repair. The poor father-child relationship will bring about a negative impact on the emotional regulation of adolescents. When it becomes difficult to regulate negative emotions, they can easily cause depression. The study reveals that the risk of depression can be effectively reduced by improving the relationship between fathers and children and improving the self-efficacy of emotional regulation.

### The Moderating Role of Gender

The study found that gender only regulates the path between NEG and adolescents’ depression. When the conflict between fathers and children increases, adolescents are more likely to experience declines in RESE, and depression will increase. However, female students are more likely to be affected by NEG than male students. When NEG is decreased, female students are more likely to suffer depression than male students, which is partially consistent with the Hypothesis 3. First, the study found that female students’ NEG is significantly lower than that of male students, whereas there is no significant difference between POS of male and female students. Because depression is often related to the individual’s cognition and ability to regulate their negative emotions, if these cannot be vented this can easily cause depression symptoms. Whether the positive emotions are expressed in a proper manner has little to do with the internal and external problems (Caprara et al., 2010). This can also explain why gender had no moderating effect on the direct prediction of POS on depression. Meanwhile, what strategy the individual employs to regulate emotions is susceptible to RESE (Tang et al., 2010). In the process of emotional regulation, males are better at adopting cognitive reevaluation strategy, while females adopt more emotional focusing strategy, which leads to females’ stronger negative emotional susceptibility (Mak et al., 2009). Thus, females have less self-efficacy in regulating negative emotions than males, and as their negative emotional susceptibility is higher than that of males, they are more prone to develop depression.

Second, according to the gender intensification hypothesis, parents are increasingly responsible for the socialization of same-sex children after entering adolescence. Parents’ role guidance for their children is more important. Parents are more intimate with same-sex children and less intimate with children of different sex (Hill and Lynch, 1983; Laursen and Collins, 2009). Some researchers have also found that fathers tended to be more involved in their son’s upbringing during adolescence (Pleck, 1997; Rosenthal, 2010). The communication between fathers and adolescent daughters is less than that with sons (Hosley and Montemayor, 1997). Compared with the adolescent communication between fathers and daughters, father-son communication is more open and self disclosure is higher (Youniss and Smollar, 1985). The decrease in communication between fathers and daughters and hence the intimacy of the father-daughter relationship causes relationship alienation between fathers and daughters making it more difficult for them to reconcile (Liu et al., 2011), which has a negative impact on the self-efficacy in managing negative affect (NEG), and a long-term backlog of bad emotions can easily induce depression. In addition, parents have different expectations for the role of males and females according to Chinese traditional cultures, and they encourage their daughters to be dependent and obedient, and their sons to be autonomous and competitive. Being a girl in adolescence is rarely seen by fathers as a process of enhancing independence. Most fathers in China associated girls’ independence with risks and moral responsibilities, which are closely related to gender (Xu, 2016). Therefore, girls are more likely to restrain negative emotions toward their father (who has a more authoritative position than the mother in family life). This study showed that father-child conflict among girls in junior high school is significantly higher than that of boys. When there are more conflicts between fathers and daughters and negative emotions are not released, their self-efficacy in regulating negative emotions will be inevitably impacted, which increases the risk of depression.

Finally, contrary to our expectations, gender does not regulate the direct path between father-child conflict and adolescent depression. The result shows that father-child conflict is significantly associated with depression in adolescents, and it does not show significant difference due to gender differences. This may be because more conflicts between fathers and children cause higher levels of depression, which is consistent between male and female students.

In conclusion, girls are more likely to see reduced self-efficacy in regulating negative emotions in conflicts with their father which causes depression. This also partly explains why after puberty, female students have a significant increase in depression compared with male students (Angold et al., 2002; Garber, 2006; Ma et al., 2020). Therefore, in the process of preventing the depression caused by parent-child conflict, it is more important to improve the RESE of girls, as this would reduce the level of depression more effectively. Conflict interveners need to pay special attention to girls in the parent-child relationship, especially when the father-daughter conflict is serious. Interveners should focus on the regulation of negative emotions and enhance the self-efficacy of emotional regulation in order to reduce the risk of depression.

### Limitations and Implications

Although this study explored the influence of father-child conflict on adolescents’ depression, and its effect paths and group differences, thus expanding and deepening the research on adolescents’ depression, there remain some shortcomings in this study. First, the research adopted a cross-sectional research, which cannot reveal the causal relationship between variables, so the investigation of the relationship between variables can only explore relevant relations, not draw causal conclusion. Second, the relationship between parents and children perceived by adolescents at different stages of adolescence differs, such as the parent-child conflict, which shows that as they move through adolescence, the parent-child conflict increases first, and then decreases, and finally tends to stabilize (Smetana et al., 2004). Whether the effect of father-child conflict on depression also presents a dynamic relationship is unknown. Therefore, longitudinal tracking research should be undertaken in the future to further explore and determine the direct relationship and dynamic changes of the above variables. Third, the impact of Chinese culture on father-child relationship should not be neglected while interpreting the findings because the sample was recruited from China in this study. In the future, more cross-cultural studies should be carried out in order to compare the possible different impact of father-child relationship on adolescent depression. Finally, this study is based on the self-reporting of adolescents and may have a social approval effect. Future research could combine both father and mother’s views to further verify the reliability of data. The effect of the father-child attachment on adolescent depression may have a compensation effect on the father-child conflict. This study did not explore this aspect and future research could include it to further enrich and deepen the understanding of the influence of parent-child conflict on adolescent depression.

## Conclusion

Father-child conflict was found to be significantly positively associated with adolescent depression and negatively correlated with RESE and its two dimensions (POS and NEG). It was found that both POS and NEG played a partial mediating role in the relationship between father-child conflict and depression. The moderating mediating model of father-child conflict on adolescent depression was established, where gender regulates the relationship between NEG and depression. Compared to boys, girls are more affected by depression at the low level of NEG.

## Data Availability Statement

The raw data supporting the conclusions of this article will be made available by the authors, without undue reservation.

## Ethics Statement

The studies involving human participants were reviewed and approved by The Ethics Committee of the School of Education of Huazhong University of Science and Technology. Written informed consent to participate in this study was provided by the participants’ legal guardian/next of kin.

## Author Contributions

CP and JC contributed to the design of the study. HW, YaL, YoL, YW, and XZ organized and analyzed the data and wrote the different sections of the manuscript. All authors contributed to manuscript revision, read, and approved the submitted version.

## Conflict of Interest

The authors declare that the research was conducted in the absence of any commercial or financial relationships that could be construed as a potential conflict of interest.

## Publisher’s Note

All claims expressed in this article are solely those of the authors and do not necessarily represent those of their affiliated organizations, or those of the publisher, the editors and the reviewers. Any product that may be evaluated in this article, or claim that may be made by its manufacturer, is not guaranteed or endorsed by the publisher.
